# Association between plasma betaine levels and dysglycemia in patients with coronary artery disease

**DOI:** 10.1042/BSR20200676

**Published:** 2020-08-17

**Authors:** Fei Guo, Xueting Qiu, Yuanting Zhu, Zhirong Tan, Zhenyu Li, Dongsheng Ouyang

**Affiliations:** 1 Department of Clinical Pharmacology, Xiangya Hospital, Central South University, Changsha, China; 2 Institute of Clinical Pharmacology, Central South University, Hunan Key Laboratory of Pharmacogenetics, Changsha, China; 3 Engineering Research Center of Applied Technology of Pharmacogenomics, Ministry of Education, Changsha, China; 4 National Clinical Research Center for Geriatric Disorders, Changsha, China; 5 Hunan Key Laboratory for Bioanalysis of Complex Matrix Samples, Changsha Duxact Biotech Co., Ltd., Changsha, China; 6 Department of Geriatric Medicine, Xiangya Hospital, Central South University, Changsha, China

**Keywords:** betaine, CAD, cross-sectional, dysglycemia

## Abstract

**Background:** Dietary betaine intake was reported to associate with favorable profile of metabolic disorders. However, the role of circulating betaine in coronary artery disease (CAD) patients with dysglycemia is still unknown. The present study aimed to investigate the potential associations between plasma betaine levels and dysglycemia in CAD patients.

**Methods:** Total 307 subjects were enrolled in the present study with 165 CAD patients (57 with dysglycemia and 108 with normal glycemia) and 142 age- and sex-matched controls (CON). Fasting plasma betaine was detected using liquid chromatography tandem mass spectrometry.

**Results:** Plasma betaine was lower in normal glycemia CAD patients (28.29 (22.38–35.73) μM) compared with healthy controls (29.75 (25.32–39.15) μM), and was further decreased in CAD patients with dysglycemia (24.14 (20.84–30.76) μM, *P*<0.01). Betaine levels were inversely correlated with fasting glucose, glycated hemoglobin% (HbA1c), diastolic blood pressure (DBP), triglyceride (TG) and alanine aminotransferase (ALT) levels (all, *P*≤0.05). Subjects in the highest betaine tertile group had lowest frequency of CAD and dysglycemia (all, *P*<0.01). Increased betaine levels were independently associated with low risk of dysglycemia in CAD after adjustment for multiple traditional risk factors (OR = 0.04, 95% CI: 0–0.37, *P*=0.01). Furthermore, betaine had good performance at distinguishing CAD with dysglycemia from normal glycemia CAD (AUC = 0.62, *P*<0.01).

**Conclusion:** Plasma betaine levels are independently and inversely associated with dysglycemia in CAD after adjustment for multiple factors, and may be useful for risk stratification of dysglycemia in CAD.

## Introduction

Coronary artery disease (CAD) is a complex multifactorial disease which is characterized by coronary artery stenosis and insufficient blood supply. Globally, CAD is the leading cause of death and morbidity, with approximately 17 million deaths per year [1–3]. Dysglycemia, defined as the presence of type 2 diabetes (T2D) or pre-diabetes, is known to be an additional risk of CAD [4]. The insulin resistance and lipid metabolism disorders in diabetic patients were reported to promote pro-atherosclerotic effects and mechanistically involved in atherosclerosis [5–7]. More aggressive and diffuse development of atherosclerosis was found in CAD patients with diabetes, consequently, CAD patients with diabetes were shown to have worse 3-year functional outcome after treatment of CAD compared CAD patients with normal glycemia [8,9]. Exploring the deranged glycemia state is important in defining the severity of associated metabolic disorders, yet there exist few markers hereof. More than that, prevention strategies based on traditional risk factors such as glucose, obesity, hypertension and hyperlipidemia could not redeem the growing prevalence of CAD and diabetes [10–12]. Given this unfulfilled need for deeper understanding of these diseases, uncovering risk factors based on metabolic profile of CAD and dysglycemia for better risk prediction and stratification is of great importance.

Betaine is a methyl derivative of glycine which possesses many important physiological functions such as methyl group donor and osmoprotectant. Betaine can be synthesized by oxidizing choline in the mitochondria in liver and kidney through a two-step enzymic reaction [13]. Besides the synthesis *in vivo*, dietary intake plays a critical role in the betaine content of the body [14]. Betaine involved in one-carbon metabolism are essential for the methylation and synthesis of DNA [15]. The change of betaine levels may indicate disorder in nutrient and physiological status [16].

Several studies have indicated the association of dietary betaine with cardiovascular disease (CVD) risk [17,18]. Betaine is used as therapy to lower plasma homocysteine, which is tightly associated with insulin resistance and cardiovascular complications, through methylation of homocysteine and generation of methionine [19]. Not only that, low betaine concentrations have been reported in subjects with metabolic syndrome [20]. In animal models, betaine treatment was also suggested to be inversely associated with inflammation which played a critical role in inducing metabolic disorder [21,22]. However, less attention has been paid to the possible associations between circulating betaine and dysglycemia in CAD patients. Understanding the pathophysiological links between dysglycemia and circulating betaine in the context of CAD could allow for more accurate identification of high-risk patients. Thus, the present study explored the associations between plasma betaine levels and CAD patients with or without dysglycemia by utilizing a hospital-based cross-sectional study among southern Chinese cohort.

## Materials and methods

### Study population and design

#### Inclusion criteria

We randomly collected a total of 1395 blood samples from patients who were hospitalized for coronary angiography in Xiangya Hospital at Central South University from June 2014 to September 2019. A total of 165 CAD patients (CAD), aged 25–75, were included in the present study. CAD was diagnosed as ≥50% stenosis in at least one main coronary artery (determined by quantitative coronary angiogram analysis). Age- and sex-matched control group (CON, *n*=142) was an independently recruited set without known dysglycemia who underwent coronary artery CT or coronary angiography for chest pain but showed negative results. We further divided CAD cases into CAD with normal glycemia group (CAD-N, *n*=108) and CAD with dysglycemia group (CAD-D, *n*=57). Dysglycemia was defined as prediabetes (fasting plasma glucose ≥ 6.1 mmol/l or non-fasting plasma glucose ≥ 7.8 mmol/l) and diabetes (fasting glucose ≥ 7.0 mmol/l or non-fasting glucose ≥ 11.1 mmol/l or history of diabetes).

#### Exclusion criteria

Subjects with an active infection, malignancy, severe liver or renal diseases, intestinal dysfunction, gastrointestinal surgery history, choline or betaine supplementation in recent 6 months were excluded.

#### Clinical data and sample collection

Cinical information including age, sex, weight and body mass index (BMI) was retrospectively collected from each subject’s medical records. Blood samples were collected using vacutainer tubes after at least 12 h of fasting to minimize the interference from dietary betaine, and immediately frozen at −80°C until analysis.

#### Ethical clearance

All participants were informed and provided written consent. The human studies have been reviewed by the Ethical Committee of Xiangya Hospital of Central South University, and have been performed in accordance with the ethical standards laid down in an appropriate version of the 1964 Declaration of Helsinki.

### Laboratory test

#### Liquid chromatography-tandem mass spectrometry analysis

Betaine in plasma were determined by high performance liquid chromatography-tandem mass spectrometry (HPLC-MS) using D9-betaine internal standards as described previously [23]. To be brief, 30 μl plasma samples were mixed with three volumes of acetonitrile containing 10 μmol/l of D9-betaine and centrifuged for 2 min at 5800×***g*** for protein precipitation. The supernatant was analyzed after injection into a normal-phase silica column (2.1 × 50 mm, 2.7 μm) and equilibrated with 25% solution A (15 mmol/l ammonium formate, pH 3.5) and 75% solution B (acetonitrile) under isocratic elution with the flow rate of 0.6 ml/min.

#### Biochemical analysis

Fasting blood glucose (Glu), glycated hemoglobin (HbA1c), creatinine (Cr), triglyceride (TG), total cholesterol (TC), alanine aminotransferase (ALT), glutamic-pyruvic transaminase (AST), low-density lipoprotein (LDL) and high-density lipoprotein (HDL) were measured using HITACHI7170S automatic biochemical analyzer.

#### Blood pressure measurement

Systolic blood pressure (SBP), diastolic blood pressure (DBP) and heart rate (HR) were measured by A&D TM-2655 automatic blood pressure measurement device.

### Statistical analysis

Continuous data are presented as means ± standard deviations (SD) for normal distribution parameters or medians (interquartile ranges, IQRs) for non-normal distribution parameters. Categorical variables are presented as numbers and percentages. Student’s *t* test or the Mann–Whitney *U* test was used for differences evaluation between two groups. Comparison between the tertiles was performed using one-way ANOVA. The Spearman correlation analysis was used to examine the correlations between betaine and clinical variables. The association of betaine with risk of dysglycemia in CAD patients was evaluated by logistic regression analysis, adjustments were made for variables including: age, gender, BMI, Glu, Cr, TG and LDL. The concentrations of betaine were log-transformed before logistic analysis. The area under the receiver operating characteristic (ROC) curve was calculated to test the accuracy of betaine at discriminating CAD with normal glycemia from CAD with dysglycemia. Best cut-off value was calculated using the maximum sum of sensitivity and specificity. A two-tailed *P*-value <0.05 was considered statistically significant.

## Results

### Patient characteristics and correlations with plasma betaine

**Figure 1:**
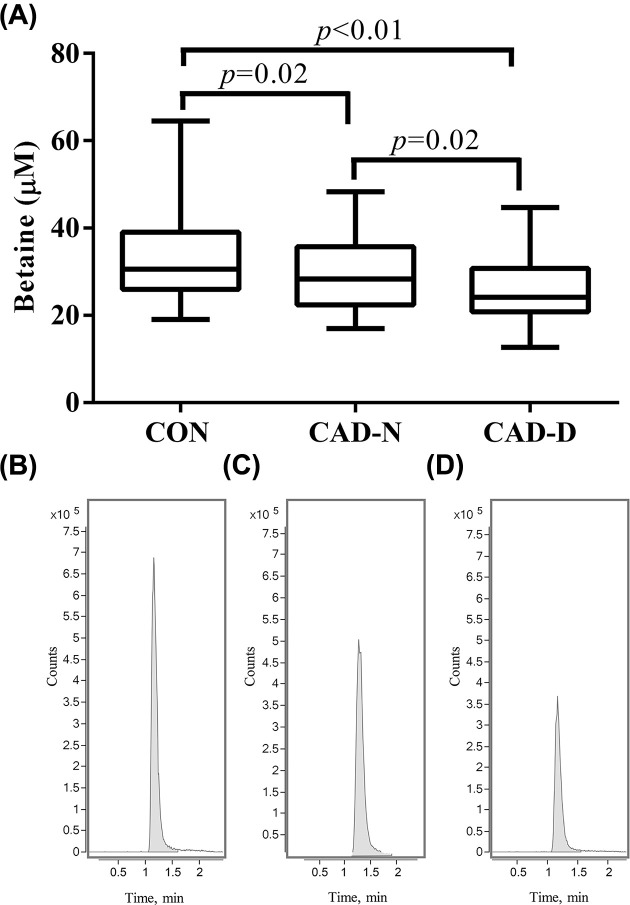
Comparison of plasma betaine levels in CON, CAD-N and CAD-D groups (**A**) Comparison of plasma betaine levels in different groups. (**B**) MS spectrogram of betaine in CON. (**C**) MS spectrogram of betaine in CAD-N. (**D**) MS spectrogram of betaine in CAD-D. Plasma betaine levels were lower in normal glycemia patients compared with healthy controls, and further decreased in CAD patients with dysglycemia compared with those without.

The general clinical characteristics of all participants are shown in Table 1. Mean age of CON, CAD-N and CAD-D groups were similar (57 ± 1 vs 59 ± 1 vs 58 ± 1, *P*=0.2). A total of 40% CON, 39% CAD-N and 47% CAD-D were males (*P*=0.69). We found no significant differences in height, weight, BMI, blood pressure, HR, TC, HDL and ALT levels between different groups (*P*>0.05). Elevated Glu and HbA1c% was observed in CAD-D (Glu: 7.13 (6.15–9.08) mmol/l, HbA1c%: 7.1 (6.4–9)) group compared with CON (Glu: 5.1 (4.67–5.63) mmol/l, HbA1c%: 5.7 (5.6–5.7)) and CAD-N groups (Glu: 5.02 (4.72–5.81) mmol/l, HbA1c%: 5.(5.7–5.8)) (*P*<0.01). CAD patients with or without dysglycemia both exhibited higher Cr, TG, AST and LDL levels compared with CON group (*P*<0.05). Plasma betaine concentrations were lower in CAD-N patients than CON (28.29 (22.38–35.73) vs 29.75 (25.32–39.15) μM, *P*=0.02). Remarkably, the betaine concentrations were even lower in CAD-D patients than normal glycemia CAD patients and CON (24.14 (20.84–30.76) μM vs 28.29 (22.38–35.73) vs 29.75 (25.32–39.15) μM, *P*<0.01) (Figure 1A–D).

**Table 1:** Baseline characteristics of subjects stratified by disease status

	CON (*n*=142)	CAD-N (*n*=108)	CAD-D (*n*=57)	*P*
Age (years)	57±1	59 ± 1	58 ± 1	0.2
Male (*n*, %)	57 (40%)	42 (39%)	26 (47%)	0.69
Height (cm)	160 (156–167)	160 (155–168)	160 (158–169)	0.8
Weight (kg)	62 (55–72)	62 (55–70)	68 (59–75)	0.08
BMI (kg/m^2^)	24 (22–27)	25 (22–26)	25 (23–27)	0.16
SBP (mmHg)	126 (116–144)	130 (114–146)	133 (120–150)	0.1
DBP (mmHg)	77 (7080)	80 (69–89)	80 (69–86)	0.33
HR	69 (64–79)	72 (66–80)	70 (63–80)	0.94
Glu (mmol/l)	5.1 (4.67–5.63)	5.02 (4.72–5.81)	7.13 (6.15–9.08)	<0.01
HbA1c (%)	5.7 (5.6–5.7)	5.7 (5.7–5.8)	7.1 (6.4–9)	<0.01
Cr (μmol/l)	79 (70.23–94.03)	85.3 (73.7–98)	83.45 (73.55–98.03)	0.03
TG (mmol/l)	1.33 (0.98–1.84)	1.77 (1.11–2.34)	1.88 (1.34–2.55)	<0.01
TC (mmol/l)	4.23 (3.66–4.92)	4.55 (3.89–5.54)	4.56 (3.63–5.55)	0.11
HDL (mmol/l)	1.12 (0.93–1.36)	1.11 (0.91–1.32)	1.06 (0.86–1.22)	0.06
LDL (mmol/l)	2.57 (2.08–3.1)	2.9 (2.25–3.59)	2.81 (2.26–3.54)	0.01
ALT (U/l)	20.3 (15.3–30.5)	25.2 (15.8–39.7)	22.25 (13.88–33.08)	0.17
AST (U/l)	21.95 (18.6–28.33)	22.4 (19.6–31)	20.21 (16.28–27.25)	0.03
Betaine (μM)	29.75 (25.32–39.15)	28.29 (22.38–35.73)	24.14 (20.84–30.76)	<0.01

Continuous data are presented as mean ± SD or median (IQR), and categorical variables are presented as counts (%).

Next, we analyzed the correlation of plasma betaine with other traditional risk factors of CVD and diabetes and discovered inverse correlations of betaine with Glu (R = −0.17, *P*<0.01), HbA1c% (R = −0.18, *P*=0.05), DBP (R = −0.13, *P*=0.03), TG (R = −0.19, *P*<0.03) and ALT (R = −0.2, *P*<0.01) by Spearman correlation analysis (Table 2). No significant correlation was observed between betaine with other factors such as age, BMI, SBP, DBP, Cr, TC, HDL, LDL and AST.

**Table 2:** Correlation of risk factors with betaine

	Betaine	
	Spearman correlation (R)	*P*
Age	0.05	0.44
BMI	−0.09	0.15
SBP	−0.02	0.71
DBP	−0.13	0.03
Glu (mmol/l)	−0.17	<0.01
HbA1c	−0.18	0.05
Cr (μmol/l)	−0.04	0.53
TG (mmol/l)	−0.19	<0.01
TC (mmol/l)	−0.09	0.14
HDL (mmol/l)	−0.06	0.33
LDL (mmol/l)	−0.06	0.29
ALT (U/l)	−0.2	<0.01
AST (U/l)	−0.06	0.29

### Comparison of clinical characteristics in different plasma betaine tertile groups

We further categorized the total study cohort into tertiles according to distribution of plasma betaine. As shown in Table 3, participants in tertile 1 with lowest betaine levels tend to have higher fasting glucose, HbA1c, TG, TC, LDL and ALT levels than those in tertiles 2 and 3. Accordingly, we found the highest percentage of CAD and T2D in tertile 1, and the percentage of CAD and dysglycemia further decreased significantly in tertiles 2 and 3 with increased betaine concentrations (*P*<0.01). No significant difference of other clinical characteristics such as age, gender, height, weight, BMI, blood pressure, HR, Cr, HDL and AST was observed between different tertiles.

**Table 3:** General characteristics of patients by tertiles of betaine levels

	Tertiles of circulating betaine			
	Tertile 1 (<25.03 μM) *n*=103	Tertile 2 (25.03–33.67 μM) *n*=102	Tertile 3 (>33.67 μM) *n*=102	*P*
Age (years)	57 ± 1	57 ± 1	58 ± 1	0.91
Male%	36%	46%	40%	0.33
Height (cm)	160 (155–168)	162 (158–167)	160 (155–167)	0.67
Weight (kg)	65 (57–75)	62 (55.38–70)	62.5 (55–69.75)	0.4
BMI (kg/m^2^)	25 (23–28)	24 (22–26)	24 (22–27)	0.4
SBP (mmHg)	130 (116–146)	130 (120–145)	128 (113–141)	0.47
DBP (mmHg)	80 (70–88)	77 (70–84)	77 (69–81)	0.11
HR	71 (64–80)	69 (64–79)	74 (65–84)	0.2
Glu (mmol/l)	5.77 (4.84–6.85)	5.2 (4.75–6.06)	5.16 (4.58–5.83)	<0.01
HbA1c (%)	7 (5.7–8.84)	6.13 (5.7–7.1)	5.9 (5.7–6.74)	0.05
Cr (μmol/l)	83.6 (71.7–95.8)	83.2 (73.6–97.3)	81.1 (75.75–96.15)	0.57
TG (mmol/l)	1.92 (1.3–2.79)	1.41 (1.01–2.05)	1.36 (1.04–1.85)	<0.01
TC (mmol/l)	4.62 (3.88–5.44)	4.27 (3.62–5.29)	4.23 (3.87–5.04)	0.03
HDL (mmol/l)	1.14 (0.9–1.33)	1.06 (0.92–1.26)	1.1 (0.93–1.34)	0.15
LDL (mmol/l)	2.9 (2.26–3.55)	2.65 (2.08–3.19)	2.61 (2.21–3.24)	0.05
ALT (U/l)	23.4 (17.8–42.05)	22.5 (14.9–32.9)	21.2 (14.55–30.8)	<0.01
AST (U/l)	21.2 (18.25–31.8)	23.4 (19.1–29.85)	22.5 (19.05–27.55)	0.08
CAD %	69%	51%	41%	<0.01
Dysglycemia %	30%	18%	8%	<0.01

Continuous data are presented as mean ± SD or median (IQR), and categorical variables are presented as percentage.

### Association and diagnostic value of betaine levels with dysglycemia

Based on logistic regression analysis, elevated betaine levels were independently associated with low risk of dysglycemia in CAD patients unadjusted (OR = 0.05, 95% CI: 0.01–0.37, *P*<0.01, model 1) or adjusted for traditional risk factors including age, gender, BMI, Cr, TG and LDL levels (OR = 0.04, 95% CI: 0.01–0.37, *P*=0.01, model 2). When further adjusted for Glu, betaine remained to be a protective role in CAD with dysglycemia but had no statistical significance (OR = 0.59, 95% CI: 0.03–13.46, *P*=0.74, model 3) (Table 4).

**Table 4:** Association of betaine levels with dysglycemia in CAD patients

	Model 1			Model 2			Model 3		
	OR	95% CI	*P*	OR	95% CI	*P*	OR	95% CI	*P*
Betaine	0.05	0.01–0.37	<0.01	0.04	0–0.37	0.01	0.59	0.03–13.46	0.74

Odds ratio shown were for plasma betaine in CAD patients with or without dysglycemia. Model 1: unadjusted. Model 2: adjusted for age, gender, BMI, Cr, TG and LDL. Model 3: adjusted for all factors in model 2 plus Glu.

**Figure 2:**
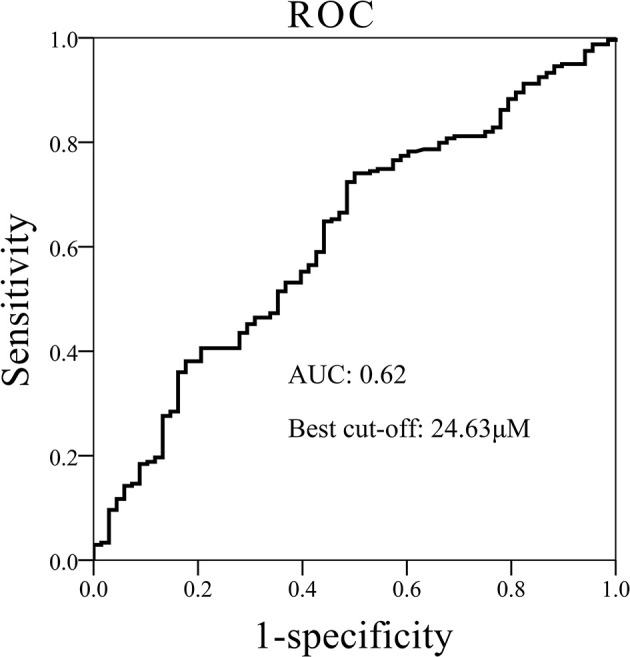
ROC curves of betaine for discriminating normal glycemia CAD from CAD with normal glycemia AUC indicated area under the ROC curve. For the best cut-off value of 24.63 μM, the likelihood of correctly predicting an event was up to 62% (AUC).

We performed ROC curve analysis to explore the diagnostic role of betaine in diabetes. As shown in Figure 2, the AUC was 0.62 and the best cut-off threshold of betaine was 24.63 μM in discriminating CAD with dysglycemia from normal glycemia CAD.

## Discussion

This is the first study to demonstrate that circulating betaine levels are inversely associated with both CAD and the risk of dysglycemia in CAD. In the present study, subjects diagnosed with CAD had lower betaine levels than CON group (Table 1). In addition, we observed further decreased betaine in CAD patients with dysglycemia compared with normal glycemia CAD patients (Figure 1A–D). Plasma betaine levels were inversely correlated with fasting glucose (Table 2). Consequently, subjects with higher betaine exhibited less frequency of CAD and dysglycemia (Table 3). These results suggested that betaine levels might be used as a potential biomarker for CAD as well as CAD with dysglycemia, further studies are needed for validation on mechanisms in betaine-related metabolic pathway.

Contradictory results were reported with regard to betaine in CVD. Higher dietary betaine intake was reported to be associated with a nonlinear higher risk of incident CAD and increased cardiometabolic mortality [24,25]. Results from Wang et al.’s study based on stable CAD participants found that plasma betaine levels were significantly higher in CAD cases than in healthy controls and there were dose-dependent associations with betaine concentration and the presence of CVD [26]. However, a recent metabolomics study revealed opposite conclusions by showing no significant association between plasma betaine and incident atrial fibrillation in the population-based Framingham Study [27]. Another study indicated that betaine supplementation could attenuate atherosclerosis in mice model [28]. In the present study, we not only found decreased betaine levels in CAD cases compared with healthy controls (Table 1), but also remarkably increased incidences of CAD along with up-regulated TG, TC and ALT levels in lower betaine tertiles compared with higher betaine tertiles (Table 3). It is well documented that rising TG and TC represents worse lipid metabolic profile and ALT levels are commonly elevated in acute coronary syndrome [29]. Taken together, our results suggested a negative correlation of plasma betaine levels with CAD risk. Notably, the median concentrations of betaine in CAD patients enrolled in Wang et al.’s study was higher than patients enrolled in the present study (41.2 vs 26.83 μM). These differences may be caused by multiple characteristics between populations such as age structure, criteria of CAD patients, the Eastern and Western dietary habits and polymorphism of key metabolic enzyme. Further studies which involve all those factors are needed for more information.

Dysglycemia-induced chronic inflammation has been associated with incident T2D and microvascular complications [30]. CVD patients with complication of diabetes have worse metabolic profile and increased mortality comparing with simple CVD patients [31]. Therapeutic approaches to diabetes management may differentially affect dysglycemia and CVDs. Thus, elucidating the potential mechanism and identifying potential biomarkers for risk stratification of dysglycemia in CAD is of great clinical importance for disease early diagnosis and prevention. Several studies have revealed the decreased betaine in patients with diabetes than those without [32,33]. So far, no study has discussed the association of circulating betaine with the presence of dysglycemia in CAD. We, for the first time, compared betaine levels in CAD patients with or without dysglycemia and revealed that, besides the decreased betaine in CAD compared with CON, CAD with dysglycemia had remarkably lower betaine than normal glycemia CAD (Figure 1A–D).

Betaine could inhibit cell proliferation and extracellular matrix deposition under high glucose stimulation via regulating the Akt/Erk1/2/p38 MAPK signaling pathway [34]. Results of Zhang et al.’s study indicated that betaine could ease hepatic fat accumulation and promoted mitochondrial content and activity [35]. These evidences suggested that betaine was involved in the lipid homeostasis and energy metabolism, which were key effectors in the pathogenesis of diabetes and CAD. A study based on 4336 participants suggested that betaine levels were negatively correlated with TC and TGs, which commonly up-regulated with blood glucose in dysglycemia [36]. Similarly, results of the Spearman correlation analysis in our study confirmed the inverse association of plasma betaine with fasting glucose, HbA1c% and TG (Table 2). We further compared the clinical characteristics between different betaine tertiles and revealed consistent results that subjects in tertile 3 (highest in betaine) had lowest Glu, HbA1c%, TG, TC and LDL levels compared with subjects in lower betaine tertiles (Table 3). Taken together, these results indicated the association of decreased betaine with worse glucose and lipid metabolic profile which might heighten the risk of dysglycemia.

Betaine is a major methyl donor necessary to convert homocysteine into methionine by betaine:homocysteine methyltransferase (BHMT). Increased hepatic BHMT activity was reported in diabetic rat model with a concomitant reduction in hepatic betaine concentration [37]. Besides, there were evidences indicated elevated urine excretion of betaine in diabetic patients [38]. Based on these results, we hypothesized that deceased betaine in diabetes may resulted from the accelerating transformation of betaine to dimethylglycine and urine excretion. However, more investigations involving expression and activity of BHMT and urine metabolic profile in CAD patients with or without dysglycemia are needed.

Chronic inflammation is another key effector in inducing dysglycemia as well as atherosclerosis. *In vitro* experiments demonstrated that betaine treatment could significantly inhibit NLRP3 inflammasome-related proteins and the levels of inflammatory cytokines including IL-1β [39]. More than that, long-term consumption of betaine has been shown to associate with decreased inflammatory factors [17]. Therefore, high betaine levels may play a protective role in reducing inflammation in dysglycemia and CAD patients.

We demonstrated the independent association of betaine levels with dysglycemia in CAD patients by logistic regression and suggested a protective role of high betaine levels in dysglycemia with reduced odds ratio even after adjustments of multiple traditional risk factors (Table 4). Consequently, low betaine levels might serve as auxiliary markers for evaluating dysglycemia in CAD. As confirmed in ROC analysis, betaine levels could correctly predict dysglycemia in CAD patients with a likelihood of 0.62 (best cut-off = 24.63 μM) (Figure 2). Taken together, these results indicated the clinical utility of betaine for dysglycemia risk prediction in CAD. Further studies with multicenter and larger sample size will be needed to further verify the sensitivity and specificity of betaine in evaluating dysglycemia.

The present study has several limitations. For example, it is a cross-sectional study, so we could not evaluate the future development of dysglycemia in CAD. The subjects in our study were enrolled in one center and the sample size was small. Therefore, selection bias cannot be excluded. And we did not include dysglycemia patients without CAD as a control group, so we were unable to analyze the variations of betaine between dysglycemia patients with or without CAD. Other potential confounding factors, such as nutritional status and activity of key metabolic enzyme, were not involved in the present study, which might also influence the results.

In conclusion, the current study explored the correlation of plasma betaine with CAD and dysglycemia in CAD. CAD patients had reduced betaine levels than healthy controls. Not only that, patients with even lower betaine were at more risk to develop dysglycemia. The present study suggested that plasma betaine levels were independently and inversely associated with dysglycemia in CAD, and might be useful for risk stratification of dysglycemia and CAD. Our preliminary results should be interpreted with caution and need to be further confirmed by large prospective studies.

## Data Availability

The datasets used and analyzed during the current study are available from the corresponding author on reasonable request.

## Abbreviations

ALT: alanine aminotransferase
AST: glutamic-pyruvic transaminase
AUC: area under curve
BHMT: betaine:homocysteine methyltransferase
BMI: body mass index
CAD: coronary artery disease
CAD-D: CAD with dysglycemia group
CAD-N: CAD with normal glycemia group
CON: control
Cr: creatinine
CVD: cardiovascular disease
DBP: diastolic blood pressure
HbA1c: glycated hemoglobin
HDL: high-density lipoprotein
HR: heart rate
LDL: low-density lipoprotein
NLRP3: nucleotide-binding oligomerization domain-, leucine-rich repeat- and pyrin domain-containing 3
OR: odds ratio
ROC: receiver operating characteristic
SBP: Systolic blood pressure
TC: total cholesterol
TG: triglyceride
95% CI: 95% confidence interval
